# Pragmatic trial on inhaled corticosteroid withdrawal in patients with COPD in general practice

**DOI:** 10.1038/s41533-020-00198-5

**Published:** 2020-10-09

**Authors:** Lisette van den Bemt, Lotte van den Nieuwenhof, Anne Rutjes, Victor van der Meer, Gerben Stege, Michel Wensing, Martina Teichert, Tjard Schermer

**Affiliations:** 1 Department of Primary and Community Care, Radboud University Medical Center, Radboud Institute for Health Sciences, Nijmegen, The Netherlands; 2 Budel Medical Centre, Budel, The Netherlands; 3 Department of Pulmonology, St. Anna Hospital, Geldrop, The Netherlands; 4 Department of General Practice and Health Services Research, Heidelberg University Hospital, Heidelberg, Germany; 5 Department of Clinical Pharmacy and Toxicology, Leiden University Medical Centre, Leiden, The Netherlands; 6 Gelre Hospitals Apeldoorn, Apeldoorn, The Netherlands

**Keywords:** Therapeutics, Chronic obstructive pulmonary disease, Clinical trial design

## Abstract

The therapeutic value of inhaled corticosteroids (ICSs) for COPD is limited. In published RCTs, ICS could be withdrawn in COPD patients without increasing exacerbation risk when bronchodilator treatment is optimized. Here we report on the feasibility and risks of ICS withdrawal in Dutch general practice for COPD patients without an indication for ICSs. In our pragmatic trial, general practitioners decided autonomously which of their COPD patients on ICS treatment could stop this, how this was done, and whether additional bronchodilator therapy was needed. We recruited 62 COPD patients (58 analysed) who were eligible for ICS withdrawal in 79 practices. In 32 patients (55.2%, 95% CI: 42.5–67.3%) ICS was withdrawn successfully, 19 (32.8%, 95% CI: 22.1–45.6%) restarted ICS treatment within six months, 12 patients (20.7%, 95% CI: 12.3–32.8%) had a moderate exacerbation, and one patient had a severe exacerbation. ICS withdrawal was successful in just over half of the patients with COPD without an indication for ICS.

## Introduction

Chronic obstructive pulmonary disease (COPD) is an umbrella term for respiratory diseases which have persistent airflow obstruction that is not fully reversible in common^1^. The global prevalence of COPD among people aged ≥30 years was 11.7%, with an estimated 384 million COPD patients globally^2^. The cornerstones of pharmacotherapy in COPD are short-acting and long-acting bronchodilators (beta2 agonists, muscarine antagonists)^1,3^. Inhaled corticosteroid (ICS)–long-acting beta2 agonist (LABA) combinations are recommended for COPD patients with frequent exacerbations despite optimal bronchodilation or concomitant asthma^1^. Between 4.4 and 38.4% of primary care COPD patients have concomitant asthma^4–6^. For these patients, ICS can be used to treat the underlying bronchial inflammation and thus the rationale for this treatment is clear.

On average, 56% of COPD patients in primary care studies have zero, and 22% have two or more exacerbations per year^7^. Exacerbation risk can be reduced by ICS in COPD patients with moderate-to-very-severe airflow obstruction and recurrent exacerbations^8^. However, ICS use is not without risks and is associated with higher prevalence of pneumonia, diabetes mellitus, osteoporosis, oropharyngeal candidiasis, and hoarseness^8,9^. Despite the limited indication of ICS in COPD, high rate of use (approximately 70% of COPD patients) has been reported for several European countries, including Switzerland, Greece, and the UK^10–12^. A recent study in the Netherlands showed that 51% of COPD patients without signs of asthma in general practices used ICS^13^. A budget impact analysis predicted a 5-year reduction of 84 million euro for the Netherlands alone where ICS overtreatment was avoided^14^.

To reduce overtreatment and its impact on patients and healthcare budgets, withdrawal of ICS in COPD patients who have been stable for a prolonged period of time and do not have features of asthma should be considered. A systematic review of published controlled withdrawal trials concluded that there is no evidence that withdrawing ICS in COPD patients results in important deterioration of patient outcomes^15^. Large randomized controlled studies showed that optimized bronchodilator therapy was as good as or even better in preventing exacerbations compared to ICS^16,17^. However, these studies differed from daily general practice care as the studies were strictly controlled, blinded, used placebos, included COPD patients with more severe disease than most patients seen in primary care, and used withdrawal procedures that are impractical in daily general practice care. Therefore, the aim of our study was to investigate whether ICS withdrawal in general practice for COPD patients without an indication for ICS is feasible and without adverse health effects for the patients involved.

## Results

### Patient recruitment and characteristics

**Fig. 1:**
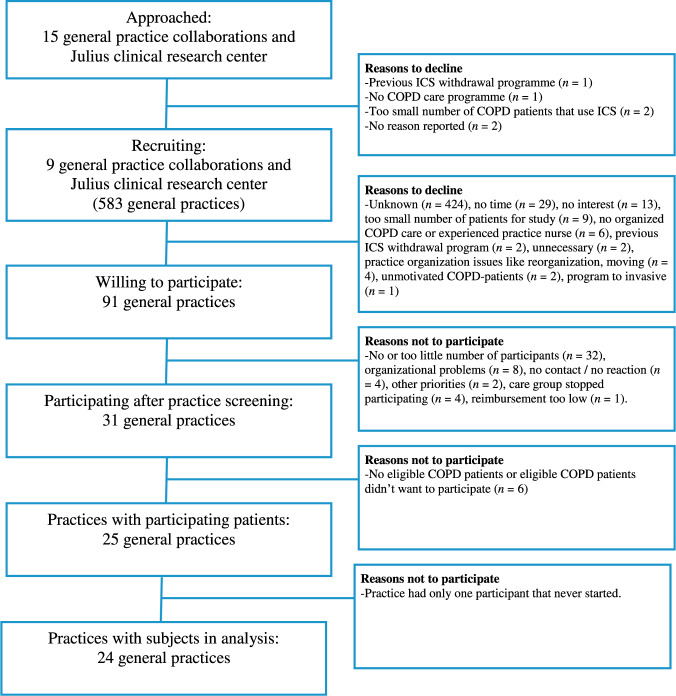
Flow chart of recruitment of general practices including reasons for non-participation. ICS inhaled corticosteroids.

Ninety-one general practices were willing to participate but ultimately only 25 practices were able to recruit one or more study participants (Fig. 1 and Supplementary Table 1). The lack of eligible patients was the main reason for practices to drop out (*n* = 32). An average of 18.3% of COPD patients in these practices were using ICS (i.e. 16 per practice); only 1 in 4 (27.3%) was considered by the general practitioner (GP) to be eligible for ICS withdrawal.

Ultimately, 62 patients participated and 2 were excluded from the analysis; 1 patient did not have COPD according to the medical record and 1 never quitted ICS. Moreover, data on the primary outcome were missing for two patients and for three patients we could not report on one or more secondary outcomes. Table 1 shows the characteristics of the 58 patients in the primary outcome analysis.

**Table 1:** Characteristics of patients with primary outcome (*n* = 58).

	*n*^a^	
Age, years (mean)	58	67.1 (SD 10.3)
Males (*n*, %)	58	29 (50.0)
Education level (*n*, %)		
Low		37 (63.8)
Medium		12 (20.7)
High		7 (12.1)
Unknown		2 (3.4)
Smoking history (*n*, %)	58	
Never smoked		3 (5.2)
Previous smoker		32 (55.2)
Current smoker		23 (39,7)
Packyears (*n*, %)	52	
≤25 packyears		25 (43.1)
>25 packyears		27 (46.6)
Inhaled corticosteroid inhaler (*n*, %)	58	
Single therapy		7 (12.1)
Combihaler with long-acting bronchodilator		51 (87.9)
Type of inhaled corticosteroids (*n*, %)	58	
Fluticasone proprionate		32 (55.2)
Budesonide		22 (37.9)
Beclometasone		4 (6.9)
Other medication used before the start (*n*, %)	58	
None (only ICS)		2 (3.4)
SABA		1 (1.7)
LABA		22 (37.9)
SABA + LABA		3 (5.2)
SAMA + LABA		4 (6.9)
LABA + LAMA		19 (34.5)
LABA + LAMA + SABA		7 (12.1)
MRC score	57	
1		28 (49.1)
2		20 (35.1)
3		7 (12.3)
4		2 (3.5)
5		0
Asthma characteristics (*n*, %)		
Diagnosed with asthma in the past	58	17 (29.3)
Asthma diagnosed in first-line family member	58	21 (36.2)
Hay fever	58	9 (15.5)
Atopic eczema	58	11 (19.0)
Relevant allergy (inhalation allergens)	57	6 (10.5)
Lung function (mean)		
Airflow obstruction (FEV1/FVC ratio)	51	0.57 (SD 0.10)
FEV1 % predicted^b^	56	64.5 (SD 13.4)
Reversibility FEV1 ≥12% (*n*)	22	2
Comorbidity (*n*, %)	58^c^	
Fatigue		13 (22.4)
Diabetes		7 (12.1)
Musculoskeletal disorders like low back pain		16 (27.6)
Osteoarthritis		17 (29.3)
Cancer		2 (3.4)
Heart diseases		8 (13.8)
Other diseases		10 (17.2)

*ICS* inhaled corticosteroid, *SABA* short-acting beta2 agonist, *LABA* long-acting beta2 agonist, *SAMA* short-acting muscarinic antagonist, *LAMA* long-acting muscarinic antagonist, *FEV1* forced expiratory volume in 1 s, *FVC* forced vital capacity.

^a^Number of participants.

^b^Based on GLI reference values.

^c^Tick boxes used, missing values unknown.

### Successful ICS withdrawal

In most cases (93.5%), ICS treatment was withdrawn immediately. In only four patients, an ICS step-down scheme was used. Twenty-six (26) patients used a LABA, 20 used a long-acting muscarinic antagonist (LAMA), and 8 used a combination of LABA and LAMA at the start of ICS withdrawal. Four patients did not receive any type of long-acting bronchodilator therapy after ICS withdrawal.

**Fig. 2:**
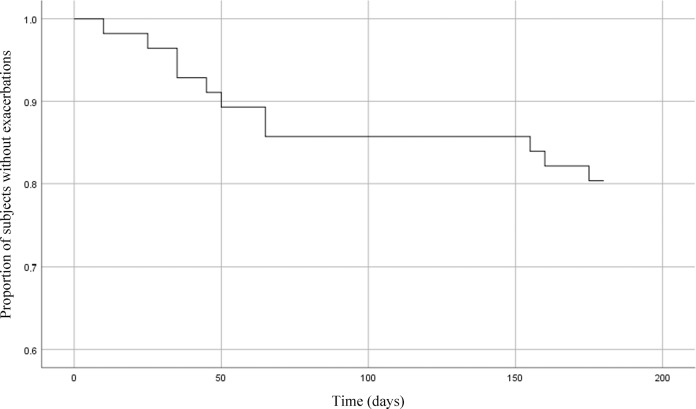
Time (days) to exacerbation after ICS withdrawal in COPD patients. Survival curve of the proportion of COPD patients that had no moderate to severe exacerbations during follow-up after inhaled corticosteroid withdrawal (*n* = 58).

**Fig. 3:**
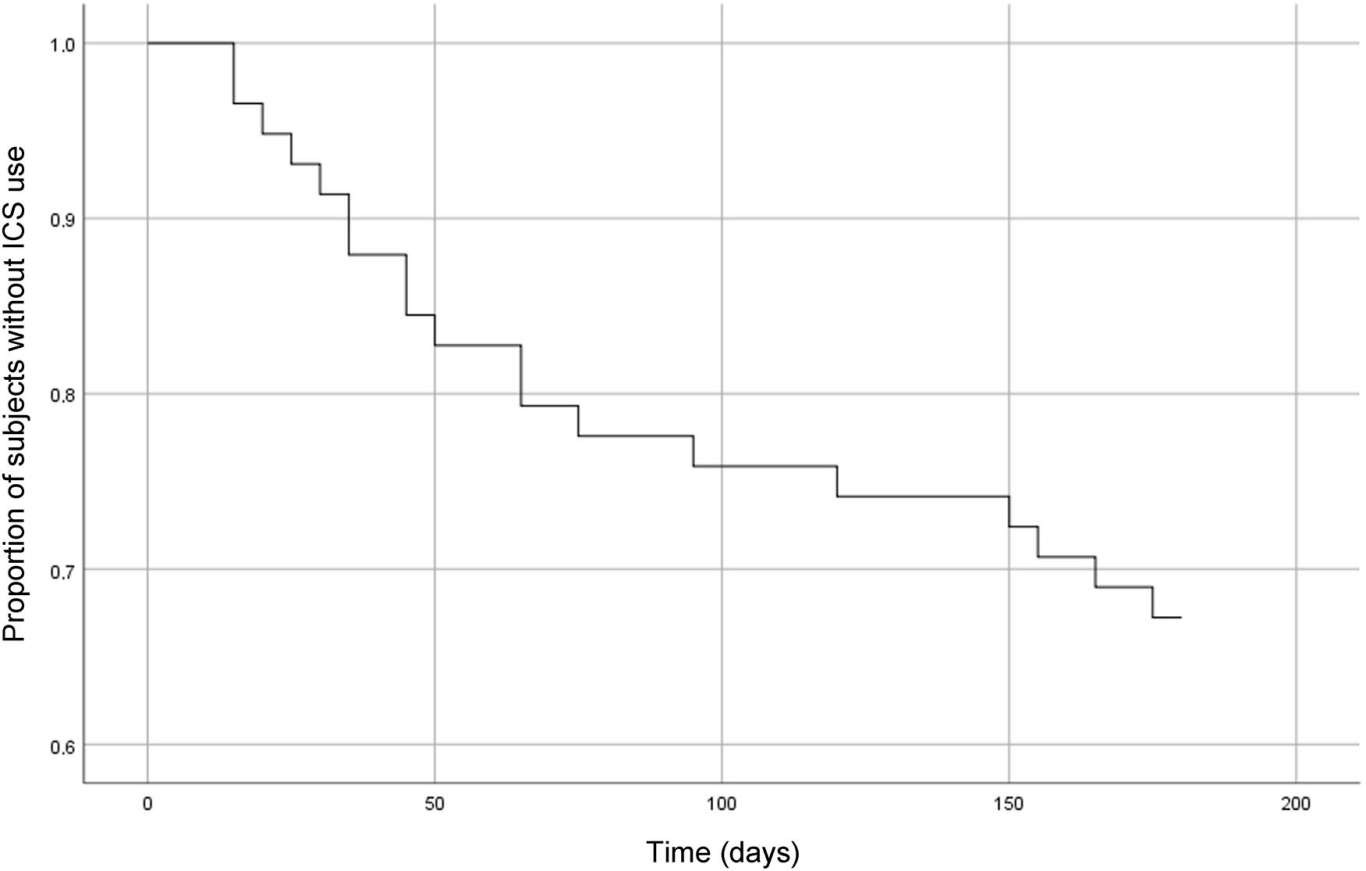
Time to restart ICS use after ICS withdrawal in COPD patients. Survival curve of the proportion of COPD patients that had no reintroduction of inhaled corticosteroids (ICS) after ICS withdrawal (*n* = 58).

Thirty-two patients (55.2%, 95% confidence interval (CI): 42.5–67.3%) were successful in ICS withdrawal; 19 (32.8%, 95% CI: 22.1–45.6%) of whom restarted ICS treatment within 6 months; 12 (20.7%, 95% CI: 12.3–32.8%) received a course of oral corticosteroids, antibiotics, or both for an exacerbation; and 1 patient was hospitalized because of an exacerbation (i.e. 22.4% of patients had a moderate or severe exacerbation). One patient had two moderate exacerbations, and therefore the total number of moderate and severe exacerbations during follow-up was 14. The median time to reintroduction of ICS treatment or a first exacerbation was 49.0 days (25–75%: 27–117) and 48.5 days (26–156), respectively (Figs 2 and 3). Two patients had an episode of pneumonia.

### Health status and dyspnoea

At baseline, only 5 patients (9.3%) scored high on side effects of ICS. Therefore, we did not further analyse changes in Inhaled Corticosteroids Questionnaire Short form (ICQ-S) scores in our study population. In Table 2, we report on health status outcomes and dyspnoea at the start and after 26 weeks of follow-up. Approximately one in four patients experienced a clinically important deterioration in these outcomes at 26 weeks compared to baseline values. The number of patients who experienced a clinically important improvement was very similar (see Table 2). Only the EuroQol Visual Analogue Scale (EQ-VAS) was statistically significant lower after 26 weeks (mean change −3.89). We compared the outcome for patients with successful ICS withdrawal to those who were unsuccessful, and the differences were neither clinically important nor statistically significant (Table 3).

**Table 2:** Change in health status, health-related quality of life, and dyspnoea of patients between baseline and 26-week follow-up^a^.

	*n*	Score baseline	Score 26 weeks	Clinical important improvement at 26 weeks	Clinical important deterioration at 26 weeks
CCQ total	55	1.21	1.23	15 (27.3)^b^	14 (25.5)
CCQ emotions	57	0.37	0.40	12 (21.1)^b^	14 (24.6)
CCQ functional status	58	1.02	1.07	14 (24.1)^b^	19 (32.8)
CCQ symptoms	57	1.84	1.84	17 (29.8)^b^	14 (24.6)
CRQ-SF	57	5.71	5.62	13 (22.8)^c^	14 (24.6)
MRC score	57			11 (19.3)^d^	12 (21.1)
1		28 (49.1)	25 (43.9)		
≥2		29 (50.9)	32 (56.1)		
EQ5D					
Index score		100 (81–100)	89 (78–100)		
VAS score	54	80 (70–88)	74 (60–80)^e^		

^a^Change in score for CCQ and CRQ-SF were tested using a paired *t* test, MRC score by McNemar, and EQ5D scores by Wilcoxon signed-rank test.

^b^Clinical important improvement is −0.4, clinical important deterioration is 0.4.

^c^Clinical important improvement is 0.55, clinical important deterioration is −0.55.

^d^Clinical important improvement is −1, clinical important deterioration is 1.

^e^Statistically significant (*p* = 0.04).

**Table 3:** Difference in secondary outcomes between patients with successful and unsuccessful ICS withdrawal^a^.

	Successful ICS withdrawal		Unsuccessful ICS withdrawal	
	*n*	∆score^b^	*n*	∆score^b^
CCQ total^c^	30	−0.06	22	0.05
CCQ emotions^c^	31	−0.05	22	0.09
CCQ functional status^c^	31	−0.06	23	0.13
CCQ symptoms^c^	30	−0.05	24	−0.02
SFCRQ^d^	31	−0,17	24	−0.08
EQ5D-3L	31	.	23	
Index score		−0.01		−0.04
VAS score		−2.29		−6.04
MRC change				
≥1 point improvement		6 (20.0%)		4 (16.0%)
0 point	30	14 (46.7%)	25	19 (76.0%)
≥1 point deterioration		10 (33.3%)		2 (8.0%)

^a^Differences in change of CCQ, CRQ-SF, and EQ5D-3L scores for successful versus unsuccessful ICS withdrawal were tested using linear regression with correction for baseline values. Change in MRC score was tested with Cronbach alpha; no result was statistically significant.

^b^The differences were neither clinically important nor statistically significant.

^c^Minimal clinical important improvement = −0.4.

^d^Minimal clinical important improvement = 0.55.

### Safety information

Apart from the one hospitalization for an exacerbation, three other patients were admitted to the hospital. These hospital admissions were neither related to ICS withdrawal nor to COPD.

## Discussion

We investigated whether ICS withdrawal in COPD patients without an indication for ICS was feasible and without adverse health effects in the general practice setting. Only 27.3% of COPD patients who used ICS were eligible for withdrawal of this treatment according to their GP. We found that almost half (44.8%) of the patients who stopped ICS either started using ICS again within 26 weeks and/or were treated for an exacerbation. Discontinuation of ICS treatment resulted in a decline in EQ-VAS. None of the other secondary outcomes changed significantly and no statistical differences in outcomes between patients with successful and unsuccessful ICS withdrawal attempts were found.

Several randomized clinical trials and observational studies on ICS withdrawal have previously been published^17–25^. Most of these studies included patients with moderate-to-very severe airflow obstruction only^17–19,21,22^. ICS withdrawal usually had no effect on exacerbation rate when bronchodilator therapy was optimized^17–20,24,25^. The use of a LABA–LAMA combination even resulted in less moderate-to-severe exacerbations compared to a LABA–ICS combination and time to exacerbation was prolonged as well^17^. One primary care study compared ICS use to placebo and found that ICS withdrawal in primary care resulted in an increased risk of exacerbations^23^. However, these patients did not receive optimal bronchodilator therapy. In another study, the effect of ICS–LABA on exacerbations did not differ from LABA alone in COPD patients with moderate airflow obstruction^24^. An observational study that included a COPD population comparable to general practice found no association between ICS withdrawal and more exacerbations compared to patients who continued to use ICS^25^. However, this study compared exacerbation risk in patients who stopped ICS use as recommended by their physician with patients who were not asked or willing to quit ICS and are therefore less comparable. The OPTIMO study showed most similarities with our design^20^. In this trial, pulmonologists were asked to include COPD patients who could quit ICS use. In all, 26% of patients who stopped ICS had at least one exacerbation within 6 months (which is comparable to the 22.4% in our study) and 29% of subjects who continued their ICS use had at least one exacerbation. In other primary care cohort studies, the 1-year prevalence of exacerbations after ICS withdrawal was even higher^7^. However, more than one moderate exacerbation in the previous year (i.e. an important risk factor for future exacerbations) was an exclusion criterion in our trial, so we anticipated a lower exacerbation rate. Overall, previous studies found that ICS withdrawal did not result in a higher risk of exacerbations when bronchodilator therapy is optimized. Our results seem to be in line with these results. More frequently, ICS withdrawal was unsuccessful because the GP decided to restart ICS use within 6 months because the patient experienced an increase in respiratory symptoms.

The EQ-VAS was statistically significantly lower at 26 weeks. We did not foresee this result, especially as the changes in disease-specific health status outcomes were much smaller. A previous study found that a change in EQ-VAS of 6.9 points (6.5–8) was a clinical relevant difference in outpatient COPD patients^26^. The change in EQ-VAS of our study population was smaller (−3.9 points) and not significantly different for successful and unsuccessful ICS withdrawals. So, this finding is most likely to be a false-positive result due to numerous outcomes and a small sample size.

According to the literature, overtreatment with ICS in COPD is widespread. Up to 70% of COPD patients across various European studies have been reported to use ICS^10–12^. In London general practices, 38% of COPD patients were overtreated with ICS according to the GOLD report^27^. Therefore, we assumed that a pragmatic clustered–randomized non-inferiority trial on ICS withdrawal in general practice with 620 patients and 2 co-primary outcomes (rate of successful ICS withdrawal and non-inferiority in the number of symptom-free weeks) was feasible (see clinicaltrial.gov NCT02691988). However, ICS use of COPD patients was considerably lower in our study in comparison to previous estimates and only one in every four patients was considered to be eligible for ICS withdrawal by their GP. Therefore, we were unable to recruit enough participants and had to reconsider our study design after a year. We decided that answering the research question on successful ICS withdrawal rate alone was still relevant and did more justice to the effort of practices and participating patients than terminating the project. The medical ethics review board endorsed our decision. One possible explanation for the low number of patients who according to their GP could quit ICS is the higher interest in ICS withdrawal of practices that were willing to participate. Moreover, there could be some degree of social desirability bias in these self-reported figures by the GPs. Multiple factors may have contributed to the low success rate of ICS withdrawals. GPs had to decide which patients could quit ICS. A ‘wrong decision’ can result in an exacerbation, which might be an important reason for GPs to be reluctant in selecting patients for ICS withdrawal, even when there is no clear indication for this treatment. Moreover, patients might be unwilling to quit ICS, as medication changes occur regularly in the Netherlands imposed by changing preference policies of health insurance companies and patients may become reluctant to change their medication again. Next, patients may link their current prescribed medication to reduced disease burden as ICS medication is usually prescribed in a period of increased symptoms. We advised the GPs to optimize bronchodilator treatment by adding a LAMA and/or LABA when ICS was stopped, but this recommendation was not always followed. For instance, four patients were only prescribed short-acting (rescue) medication after their ICS was stopped, although this may have been a deliberate choice made by their GPs because they deemed short-acting bronchodilator treatment to be sufficient for these patients. In addition, it can be disputed whether all reintroductions of ICS were necessary. In some cases, only a mild change in symptoms may have resulted in the reintroduction of ICS. Precaution from the patient’s or GP’s side may well have played an important role in these decisions as well.

In our trial, we gave participating GPs room to implement ICS withdrawal based on evidence and personalized medicine principles. GPs and practice nurses received education, decision aids, and materials to implement guided ICS withdrawal in their COPD patients. Still, this approach was not always successful, as not all selected patients turned out to be eligible for ICS withdrawal (for example, some patients had reversible airflow obstruction), bronchodilator treatment was not always optimized, and the use of the exacerbation action plan was not clear for at least one patient (who thought it was a questionnaire and sent it to the research team). Next, some practices were willing to participate but were unable to do so due to high workload in the practice. One option that might help ICS withdrawal in general practice is to offer more guidance to the GPs. For example, the first results on virtual case reviews of COPD patients prescribed high doses of ICS in the UK were promising but need to be verified by scientific studies^28^.

Despite its limitations we believe that this study adds to the current body of knowledge on ICS withdrawal in COPD as it reflects the difficulties that occur when trying to implement a strategy that was effective in strictly controlled trials into the routine of general practice. A more effective way to reduce ICS use in COPD is a clearer policy towards first ICS prescriptions in patients who may have COPD. Twenty-one (21) percent of COPD patients (without asthma) who use ICS receive their first ICS prescription even before COPD was diagnosed^13^. As it is very difficult to reverse this policy, only patients with clear indication for ICS use should be prescribed this medication.

Blood eosinophil level is a promising biomarker to identify COPD patients in whom ICS treatment is effective in reducing exacerbations^29^. Although the SUNSET study has shown that ≥300 blood eosinophils/μl predicts exacerbation risk in patients with moderate-to-severe COPD^30^, further research is needed to verify that this is indeed the optimal cut-off value and to establish the effects of an ICS withdrawal approach based on blood eosinophil level. Next, overdiagnosis of concomitant asthma can result in an overuse of ICS. In a previous study in Dutch general practices, we found that a diagnosis of asthma could only be verified based on information in the medical record for one in three patients who were diagnosed with asthma COPD overlap^31^.

Withdrawal of ICS was successful in just over half of the patients with COPD without an indication for this anti-inflammatory treatment. Despite previous reports of substantial overtreatment of COPD with ICS in general practice, recruitment of the intended number of patients for this study turned out to be very difficult. The lessons learned from this study should be taken into account when choosing implementation strategies to reduce ICS overtreatment in COPD patients in general practice. Use of a decision tree such as the desktop helper on guidance on ICS withdrawal issued by the IPCRG (https://www.ipcrg.org/dth6) or the algorithm published by Kaplan^32^ could support GPs in reducing unnecessary treatment with ICS among patients with COPD. Future studies should look at the value of blood eosinophil levels when assessing the potential benefit of ICS treatment or its withdrawal in patients with COPD.

## Methods

### Study design

We performed a pragmatic, uncontrolled, unblinded intervention study in Dutch general practices with a follow-up of 26 weeks per patient. The study was pragmatic in nature as only limited inclusion and exclusion criteria were used, GPs decided on which patients to include, how to quit ICS treatment (i.e. with or without a dosage step-down period), and which additional bronchodilator therapy (if any) was indicated. Patients were recruited between December 2016 and July 2018. The study was approved by the medical ethics review board of the Radboud university medical center (file number 2015–1834). Patients gave written informed consent before any study procedure took place.

### Study recruitment and patients

GPs were invited to participate through general practice collaborations and by a contract research organization (Julius clinical research center, Utrecht, the Netherlands). GPs who agreed to participate compiled a list of all COPD patients aged ≥40 years in their practice who had used ICS for at least the previous 6 months without a clear indication based on the information in the electronic medical patient records and who were primarily managed for their COPD by a GP. These patients received study information, a questionnaire, and were invited for an ICS re-evaluation visit at the GP office. At the ICS re-evaluation visit, the GP checked inclusion and exclusion criteria. Patients had to have a documented airflow obstruction according to their latest spirometry test (i.e. forced expiratory volume in 1 s/forced vital capacity <0.7)^1^. Patients who had had two or more exacerbations (defined as treatment with a course of oral corticosteroids, antibiotics, or both and/or a visit to an emergency care facility or a hospital admission) in the previous 12 months were excluded. A concomitant diagnosis of asthma was also the reason for exclusion. Moreover, the GP decided whether or not signs of underlying asthmatic disease (e.g., allergy and/or respiratory symptoms that started early in life) warranted ICS continuation. Other exclusion criteria were daily oral steroid use, poor mastery of the Dutch language, and reduced life expectancy.

### ICS withdrawal

Optimal bronchodilator therapy is essential for safe ICS withdrawal. Therefore, an e-learning on optimal pharmacotherapy in COPD from the Dutch Institute for Rational Use of Medicine (https://www.medicijngebruik.nl/english) was mandatory for participating GPs (and practice nurses involved) before the first study visit.

Eligible patients were asked by their GP whether they would consider supervised withdrawal of ICS treatment, and if they agreed, to provide written informed consent for study participation. GPs were instructed to favour immediate ICS withdrawal over a dosage step-down period, unless there were reasons to choose otherwise, for instance, the patient’s reluctance to quit ICS immediately. In that case, it was recommended to use halve the dose of ICS for 2 weeks and to stop ICS completely if the patient did not experience negative effects during this period.

All patients received an exacerbation action plan. This action plan was developed to support early detection of symptom deterioration and provide knowledge on how to act when such a deterioration occurred^33^. Inhaler technique was assessed and instructed by a practice nurse. Finally, GPs and practice nurses were advised to follow-up the ICS withdrawal after 2–4 weeks in order to evaluate possible changes in health status.

### Study outcomes and measurements

Primary outcome was the proportion of patients with successful cessation of ICS, i.e. a period of 26 weeks after the withdrawal date without the occurrence of a moderate or severe exacerbation or reintroduction of ICS treatment. We considered an ICS withdrawal success rate of 80% in the study population to be clinically relevant.

GPs were instructed to fill out and submit an adverse event form in case of a moderate-to-severe exacerbation or reintroduction of ICS treatment. At the end of the study, we retrieved copies of the patients’ medical records and two authors (L.v.d.B. and A.R.) assessed the records for missed cases of exacerbations and ICS reintroductions independently. When these authors came to different conclusions, a third assessor (Joke Grootens, see Acknowledgements) was asked to review the record as well.

Secondary outcomes were the time to the first exacerbation or reintroduction of ICS, the number of moderate and severe exacerbations, and the prevalence of pneumonia. We also assessed the effect of ICS withdrawal on health status and experienced side effects of inhaled medication use. Disease-specific health-related quality of life (HRQoL) was measured using the Short-form Chronic Respiratory Questionnaire (SF-CRQ) at baseline and 26 weeks follow-up^34^. The SF-CRQ consists of 8 questions in four domains (i.e. fatigue, mastery, emotional functioning, and dyspnoea). The distribution-based minimal clinically important difference (MCID) is ≥0.55 points. COPD-specific health status was measured using the Clinical COPD Questionnaire (CCQ)^35^. The CCQ consists of ten questions in three domains: symptoms, mental state, and functional state. The MCID for the CCQ is ≥0.4 points^36^. The Medical Research Council (MRC) score was used to assess dyspnoea during daily activities^37^. Side effects of ICSs were measured with the ICQ-S^38^. The EuroQol 5 dimensions 3 levels (EQ5D-3L) was used to measure general HRQoL^39^. Dutch reference values were used to calculate the EQ5D-3L index value (EQ index).

### Statistical analysis

Sample size calculation showed that 62 patients were needed to demonstrate that at least 80% of COPD patients were able to withdraw ICS successfully with 10% margin of error and a 95% CI in a large population (>20,000). The numbers and percentages of patients who were successful in ICS cessation (i.e. no ICS restart and/or moderate-to-severe exacerbation within 26 weeks of follow-up) are reported. The 95% CI of the sample proportions were calculated using the Wilson method as recommended for small sample sizes. Median time to exacerbation and/or ICS restart are presented, including 25–75% percentiles.

Mean scores at the start and at 26 weeks of follow-up are reported for CCQ and CRQ-SF, including the numbers of patients with clinically important improvement and deterioration, respectively. Differences between baseline and follow-up scores were tested using paired *t* tests. Median EQ index and EQ-VAS with 25–75 percentiles are reported for the start and 26 weeks of follow-up, and the difference in scores was tested using Wilcoxon signed-rank test. MRC score was dichotomized in MRC score 1 and MRC score ≥2. The difference in MRC scores at baseline and at 26 weeks of follow-up was tested using McNemar test. Linear regression with correction for baseline values was used to test whether changes in CCQ and CRQ-SF scores differed between patients with successful ICS withdrawal and those with an unsuccessful attempt. We used the IBM SPSS software (Chicago, IL, USA, version 25) for all analyses and *p* < 0.05 was considered statistically significant.

### Reporting summary

Further information on research design is available in the Nature Research Reporting Summary linked to this article.

## Supplementary information

Supplementary Table 1

Reporting Summary

## Data Availability

The data that support the findings of this study are available from the corresponding author upon reasonable request.
