# Supplementary material for: Pragmatic trial on inhaled corticosteroid withdrawal in patients with COPD in general practice
Source: NPJ Prim Care Respir Med. 2020 Oct 9;30:43. doi: 10.1038/s41533-020-00198-5 (PMC7547069; doi:10.1038/s41533-020-00198-5)
Supplement: Supplementary file 1 — Supplementary Table 1 [file 41533_2020_198_MOESM1_ESM.docx]

Supplemental table 1. Subject recruitment in practices willing to participate

|  | Number of practices | Practices with recruitment information | | |
| --- | --- | --- | --- | --- |
|  | n | n | % of practices | Number of patients mean (min-max) (SD) |
| Practice population (n) | 91 | 79 | 86.8 | 4124 (1034-18000) (SD 2735) |
| COPD patients (n) | 91 | 77 | 84.6 | 91 (22-352) (SD 61) |
| Prevalence of COPD (%) | 91 | 73 | 80.2 | 2.3 (1.0-5.1) (SD 0.8) |
| COPD patients that use ICS (n) | 72 | 51 | 70.8 | 16 (2-63) (SD 13.5) |
| COPD patients that use ICS (% of COPD) | 72 | 50 | 70.4 | 18.3 (3.5-41.8) (SD 9.6) |
| COPD patients eligible for participation based on practice screening (n) | 72 | 59 | 81.9 | 4.4 (0-19) (SD 5.0) |
| COPD patients eligible (% of ICS users) | 72 | 50 | 70.4 | 27.3 (0-100) (SD 30.7) |
| COPD patients that were invited (n) | 31 | 31 | 100 | 6.9 (1-19) (SD 5.1) |
| COPD patients that participated (n) | 31 | 31 | 100 | 1.9 (0-6) (SD 1.6) |
| COPD patients that participated of practices with participants (n) | 25 | 25 | 100 | 2.6 (1-6) (SD 1.4) |
| COPD patients in analyses | 25 | 25 | 100 | 2.4 (0-6) (SD 1.5) |
